# Depletion of P2X4 receptor alleviates prostate cancer bone metastasis through reduced cancer cell invasiveness and enhanced cell adhesion activities

**DOI:** 10.1007/s11302-025-10096-5

**Published:** 2025-06-14

**Authors:** Jiepei He, Yuhan Zhou, Hector M. Arredondo Carrera, Nan Li, Alison Gartland, Ning Wang

**Affiliations:** 1 Division of Clinical Medicine, The University of Sheffield, Sheffield, S10 2RX UK; 2 Leicester Cancer Research Centre, Department of Genetics, Genomics, and Cancer Sciences, University of Leicester, Leicester, LE2 7LX UK

**Keywords:** P2X4 receptor, Prostate cancer, Bone metastasis, Cell adhesion, RNA-seq

## Abstract

Prostate cancer (PCa) preferentially metastasizes to bone, which remains incurable and contributes significantly to mortality and morbidity. The P2X4 receptor (P2X4R) is a receptor for ATP that is highly expressed in many cancer types including PCa and is positively associated with tumorigenesis. To understand the role of P2X4R in PCa biology, particularly in PCa bone metastasis, P2X4R (*P2RX4*) was knocked out in human PCa cell line PC3 cells using the CRISPR/Cas9 system. Cell proliferation, apoptosis, migration, and invasion were examined using CyQUANT, Cell Meter Caspase 3/7, scratch and transwell assays. Results showed that depleting P2X4R significantly reduced cell proliferation and invasion and increased apoptosis compared to PC3 wildtype (WT) controls in vitro. To test their metastatic potential in vivo, PC3 WT and knock-out (KO) cells were intracardiacally injected into male BALB/c immunocompromised mice. Twenty-five days post-injection, there were no detectable tumours and associated bone destruction in the tibias of mice injected with KO cells, whereas tibias of over 50% mice injected with WT cells were occupied by tumour cells, with significant bone destruction observed ex vivo using micro-CT. Furthermore, RNA-seq and bioinformatic analysis of P2X4R KO cells demonstrated links between P2X4R and PCa cell adhesion, and other key signalling such as Wnt signalling. These findings suggest that P2X4R is a potential therapeutic target for PCa metastasis, particularly bone metastasis.

**Supplementary Information:**

The online version contains supplementary material available at 10.1007/s11302-025-10096-5.

## Introduction

Prostate cancer (PCa) is the second most frequently diagnosed cancer in men worldwide, with approximately 1.41 million new cases diagnosed annually representing about 14.1% of all new cancer cases in men [1] and causing 375,000 deaths globally in 2020 [2]. PCa can metastasise to distant organs such as the liver, lungs, and brain, but preferentially to bone [3]. More than 70% of patients with advanced PCa are estimated to develop skeletal metastasis [4], with approximately 80% of men who died from the disease showing evidence of bone metastases [3]. Finding novel treatments for PCa bone metastasis, which causes significant mortality and morbidity, is a major priority in the PCa research field.

Purinergic signalling, mediated by P2 purinergic receptors, has been shown to play important roles in inflammation and cancer progression [5]. P2X receptors are a group of ATP-gated ion channels involved in various biological processes [6] with seven known mammalian members (P2X1–P2X7) [7]. Previously, P2X4R has been demonstrated to be closely related to tumorigenesis including breast cancer, gastric cancer, and importantly PCa [8–10]. We have previously showed that P2X4R was the most highly expressed P2 purinergic receptor in PC3, LNCaP, and C4-2B PCa cell lines and P2X4R antagonists had anti-tumorigenic effects in a PCa xenograft model [11]. This is consistent with observation by Maynard et al. using clinical samples and allograft models [12]. More importantly, evidence suggests a potential association between P2X4R and cancer metastasis. A recent study showed that P2X4R plays a significant role in promoting Epithelial–mesenchymal transition (EMT) and enhancing the metastatic potential of gastric cancer cells [8]. P2X4R promotes invasion, tumour growth, and metastasis in breast cancer both in vitro and in vivo. Its pro-malignant role involves the regulation of lysosome acidity, promotion of autophagy, and cell survival [9]. P2X4R’s contribution to autophagy and EMT is particularly pronounced under metabolic challenges [10]. Activation of P2X4R promotes PCa cell migration and invasion by inducing EMT, while P2X4R expression is elevated in metastatic PCa [12]. However, whether P2X4R contributes to PCa bone metastasis has not been established. This study aimed to answer this question by testing the hypothesis that P2X4R activity promotes PCa bone metastasis.

To achieve the objective, CRISPR/Cas9 was used to genetically knock out (KO) the *P2RX4* in PCa cells. The effects of P2X4R-KO on various cellular processes, including proliferation, migration, invasion, and apoptosis, were examined. Furthermore, an intracardiac xenograft mouse model was utilized to assess whether genetic KO of *P2RX4* influences PCa metastasis to the bone. Additionally, RNA-seq bioinformatics analysis was employed to gain insights into the transcriptional alterations in P2X4R-KO PCa cells.

## Materials and methods

### Cell lines and P2X4R knockout

The PCa PC3 cell line was obtained from American Type Culture Collection (ATCC, Manassas, VA, USA). PC3 cells were maintained in Dulbecco’s Modified Eagle’s Medium (Thermo Fisher), supplemented with 10% FCS (Sigma) and 1% penicillin–streptomycin (Thermo Fisher). The PC3 cell line was confirmed mycoplasma free by regular PCR based mycoplasma screening. The P2RX4 KO CRISPR/Cas9 plasmid was purchased from Santa Cruz (sc-401779-KO-2) and is a pool of 3 different gRNA plasmids (gRNA1: CTCCGTTACGACCAAGGTCA targeting exon 3; gRNA2: TCACGTTGGTCATGACGAAG targeting exon 4; gRNA3: GTGCTTGTAGGAGTCTCAAC targeting exon 6 and spanning to the adjacent intron).

PC3 cells were transfected with the P2RX4 CRISPR/Cas9 KO plasmid using Lipofectamine™ 3000. Transfected cells were screened based on transient GFP expression using a FACSMelody cell sorter and successful P2X4R KO individual clones were verified at the genomic, transcriptional, and functional levels, via Sanger sequencing, RT-PCR, and Fluo-4 Direct™ Calcium assay, respectively.

PCR primers for Sanger sequencing gRNA-targeted regions:

- SgRNA1-F: ACATCGTACTTCCAGCCACT
- SgRNA1-R: CTCGGTCCCTTAGCACAT
- SgRNA2-F: CGGTCAGTGTTTGAGTTG
- SgRNA2-R: CAGCAGTGAATGTAGAGGG
- SgRNA3-F: TAACTGTCTTCCTCCGATTC
- SgRNA3-R: GTCACCTGCACCCTTCTC

RT-PCR primers for P2X4R:

- Forward: TCATCCGCAGCCGCAAAG
- Reverse: TCATCCTCCACCGGGCACCA

### Proliferation assay

The proliferation of PC3 WT/KO cells was assessed using the CyQUANT™ NF Cell Proliferation Assay (Thermo Fisher) based on cellular DNA content and fluorescence. PC3 cells were seeded at a density of 2 × 10^3^ cells per well in a 96-well plate containing medium. At 24-, 48-, 72-, and 96-h post-seeding, the medium was replaced with 1 × dye binding solution. After 1 h of incubation, fluorescence was measured using an EnSight Multimode Plate Reader (PerkinElmer) with excitation at 485 nm and emission at 530 nm.

### Apoptosis assay

PC3 WT/KO cells were plated at a density of 1 × 10^4^ cells per well in 96-well plates and incubated in the medium for 24 h. The apoptotic activity of the cells was assessed using the Cell Meter™ Caspase 3/7 Activity Apoptosis Assay (Stratech) according to the manufacturer’s instructions. Fluorescence measurements were performed using an EnSight Multimode Plate Reader (PerkinElmer) with excitation at 490 nm and emission at 525 nm.

### Migration assay

PC3 WT/KO cells were plated at a density of 2 × 10^5^ cells per well in 24-well plates and allowed to attach overnight. Mitomycin C (5 µg/mL, Sigma) was added and incubated for 1 h to inhibit cell proliferation. Vertical scratches were then created using a 200 µl pipette tip, and the cells were gently washed three times with serum-free medium to remove any detached cells. Cells were then left for 18 h after which time images were taken using an inverted Nikon phase contrast microscope (Nikon Inc., Tokyo, Japan). The percentage of scratch closure was determined using the ImageJ software (National Institutes of Health, Bethesda, MD, USA).

### Invasion assay

Transwell® cell culture inserts, with an 8-µm pore polycarbonate filter (Sigma), were coated with 1.5 mg/mL Matrigel (Corning Life Sciences, Corning, NY, USA) and incubated for 2 h. PC3 cells were seeded at 5 × 10^4^ cells/well into the top chamber in serum-free medium, and treated with 5 µg/mL Mitomycin C, while 10% FBS was added in the lower chamber as a chemoattractant. After 72 h of incubation, the inserts were fixed in ethanol, stained with eosin and haematoxylin, and mounted on slides. Slides were scanned using the Pannoramic 250 Digital Scanner. The percentage of invaded cell area was quantified using the ImageJ software.

### Mice xenografts

Six-week-old male BALB/cAnNCrl immunocompromised (athymic nude) mice (Charles River, Kent, UK) were housed under controlled conditions in Optimice cages (Animal Care Systems, CO, USA) with a 12-h light/dark cycle at 22 °C and ad libitum access to water and a 2018 Teklad Global 18% protein rodent diet containing 1.01% calcium (Harlan Laboratories, Huntingdon, UK). Mice were randomized into two groups (10 mice per group) and intracardially injected with PC3 WT or PC3 P2X4R KO cells (1 × 10^5^ per 100µL PBS per injection), respectively. The relatively moderate KO clone (KO-1) was used for the in vivo study, in order to generate more objective data. All mice were sacrificed on day 25 post-inoculation when the first mouse reached the humane endpoint. Tibias were collected and fixed in 10% formalin. All procedures complied with the UK Animals (Scientific Procedures) Act 1986 and were reviewed and approved by the local Research Ethics Committees of the University of Sheffield under Home Office project licence PF61050A3 (Sheffield, UK).

### Micro-CT analysis

Micro-CT scanning on right tibias was conducted at the resolution of 4.3 μm, using the Skyscan 1272 scanner (Bruker, Billerica, MA, USA). The X-ray source operated at a voltage of 50 kV and a current of 200 µA, utilizing a 0.5 mm aluminium filter. Trabecular bone parameters were measured from a 1.0 mm thick region 0.2 mm below the growth plate where metastatic tumour cells are generally situated. Nomenclature and symbols were used to describe the micro-CT derived bone morphometries according to the published guidelines [13].

### Tumour burden measurement on histological sections

Dissected right tibias were subjected to decalcification using 20% EDTA solution (ethylenediaminetetraacetic acid) for a period of 4 weeks, with regular replacement of the EDTA solution every 3 days. Following decalcification, the samples were embedded in paraffin wax and sectioned into 3 μm in thickness using an RM2265 microtome (Leica, Wetzlar, Germany) until the tibia head was exposed. Following hematoxylin and eosin (H&E) staining as previously described [14], the bone sections were examined under a DMRB microscope (Leica, Wetzlar, Germany) and the OsteoMeasure7 v4.2.0.1 software (OsteoMetrics, Decatur, GA, USA) was used to manually measure the percentage of the bone marrow occupied by tumours as an index of tumour burden.

### RNA-seq and bioinformatics analysis

Total RNA was extracted from two PC3 P2X4 KO clones (KO-1 and KO-2) and three WT control cell lines, using the ReliaPrep™ RNA Cell Miniprep System (Promega). Samples with adequate RNA integrity (RNA integrity number (RIN) > 9.5) were selected for bulk RNA-sequencing. RNA library preparation and sequencing were performed by Source BioScience (Nottingham, UK). RNA-seq data can be accessed on the GEO repository (submission GSE245125). RNA-seq analysis was performed as follows. The principal component analysis (PCA) variance of samples was performed by Python (https://pypi.org/). Gene expression analysis was performed on Galaxy Europe (https://usegalaxy.eu/) with significantly differentially expressed genes being defined as logFC ≥ 1 and *p* < 0.05. Gene functional annotation and pathway analysis was performed on DAVID (https://david.ncifcrf.gov/). Molecular interaction networks analyses were performed on upregulated and downregulated gene lists, respectively, using the open-source software platform Cytoscape (https://cytoscape.org).

### Statistics

The differences between the groups were analysed using unpaired *t*-tests or one-way ANOVA tests with Bonferroni’s multiple comparisons as appropriate using GraphPad Prism 8. The results are presented as mean ± standard deviation (SD), and *p*-values less than 0.05 were considered statistically significant.

## Results

### Successful knockout of P2X4R in PCa cells

**Fig. 1:**
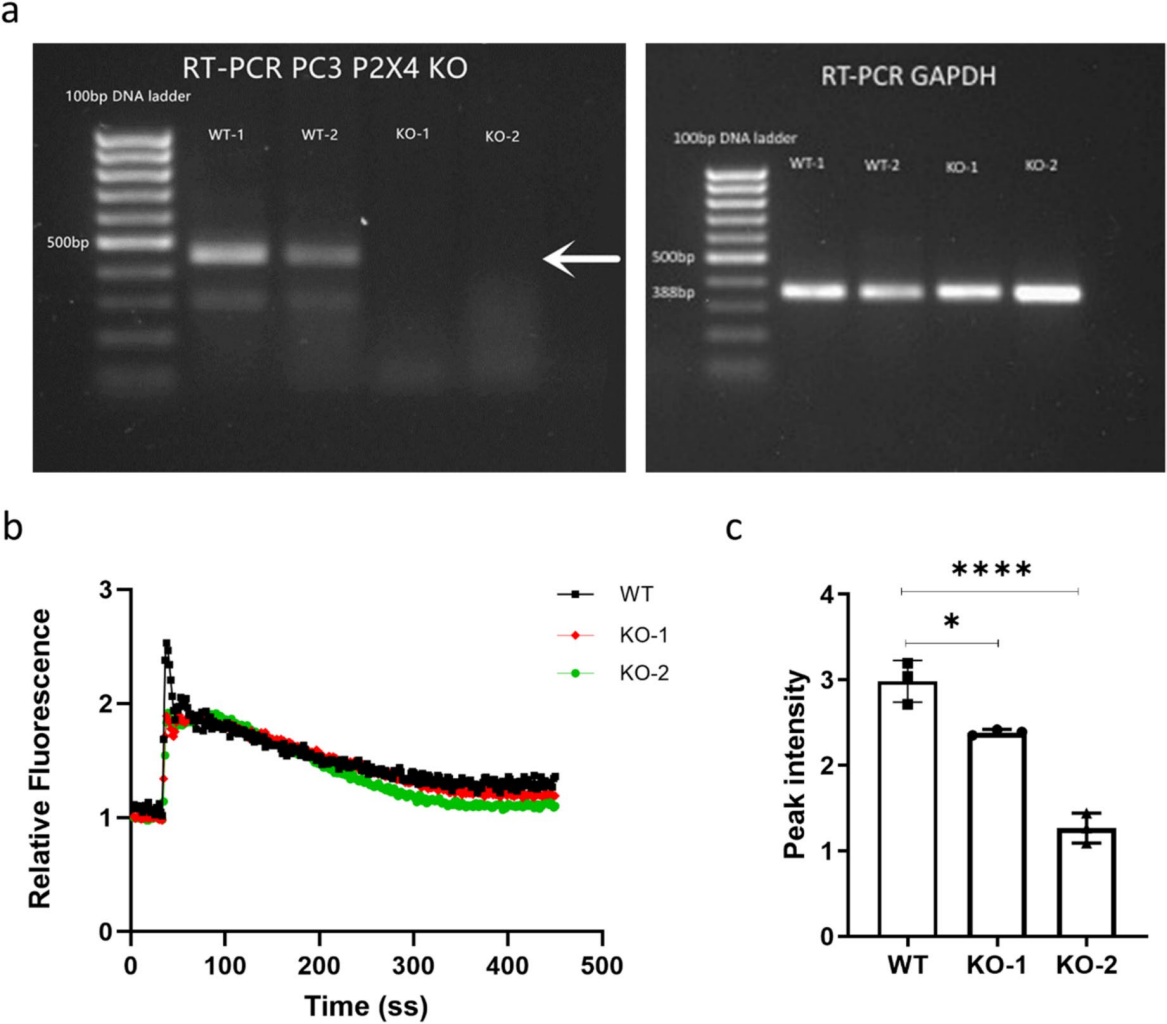
Successful KO of P2X4R in PCa cells. Following CRISPR/Case 9 KO plasmids transfection and single cells sorting using FACS based on transient GFP + signals. **a** The disruption of *P2RX4* at the RNA level of two KO clones (KO-1 and KO-2) was confirmed using RT-PCR. **b** A Fluo-4 Direct™ calcium influx detection assay was performed to examine functional changes of P2X4R in PC3 WT and KO cells stimulated with 50 μM ATP. **c** Significant reductions in peak calcium influx in both KO-1 and KO-2 cells confirmed impaired P2X4R in KO cells. Data are the mean ± SD, *n* = 3, one-way ANOVA with Bonferroni’s multiple comparisons test, *p < 0.05, ****p < 0.0001

The P2RX4 KO plasmid was transfected into PC3 WT cells using Lipofectamine 3000 transfection assay and single cells were FACS sorted based on transient GFP + signal at 48-h post-transfection. Two KO clones (KO-1 and KO-2) from single cell origins were identified with the expression of *P2RX4* being disrupted at the RNA level using RT-PCR (Fig. 1a), and confirmed via Sanger sequencing at the SgRNA 2 targeted region (Supplementary Fig. 1a and b). Furthermore, a Fluo-4 Direct™ calcium influx detection assay was performed on PC3 WT and KO cells stimulated with ATP at the concentration of 50 μM, and showed a significant reduction in peak calcium influx in PC3 P2X4R KO cells (KO-1 and KO-2). The calcium influx was significantly reduced by 20.0% for PC3 KO-1 (*p* = 0.0118), and 57.6% for PC3 KO-2 (*p* < 0.0001) respectively, demonstrating the successful functional depletion of P2RX4 in PC3 cells (Fig. 1b and c).

### Knocking out P2X4R significantly reduces PCa proliferation and invasion, and enhances apoptosis

**Fig. 2:**
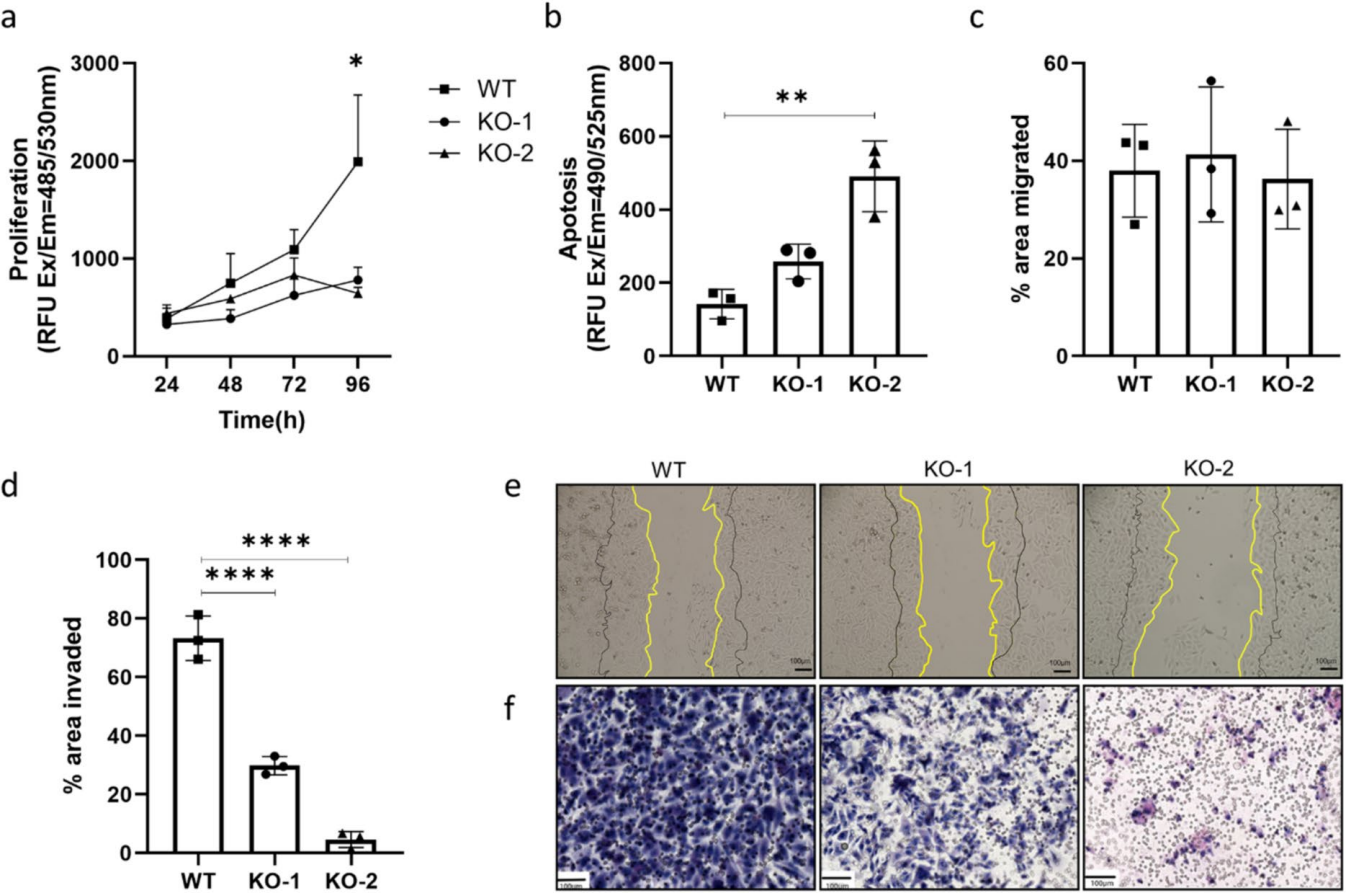
Knocking out P2X4R in PCa cells reduces cell growth and invasiveness. **a** CyQUANT cell proliferation assay was used to test cell proliferation among WT control and KO cells up to 96 h. **b** The apoptotic activity was measured using Cell Meter™ Caspase 3/7 apoptosis assay after an hour of Caspase 3/7 reagent incubation at room temperature. **c** A scratch closure migration assay was used to test the migration ability, by measuring the percentage areas of migration closure after 18-h incubation (area between the yellow and black lines). **d** Matrigel-based membrane chamber-mediated invasion assay was used over a 72-h period for testing cell invasion. **e–f** The representative images for migration or invasion of WT and KO cells. Scale bar = 100 μm. Data are the mean ± SD, *n* = 3, one-way ANOVA with Bonferroni’s multiple comparisons test, *p < 0.05, **p < 0.01, ****p < 0.0001

CyQuant proliferation assay was performed to investigate the effect of knocking out *P2RX4* on PCa cell growth. Results indicated that knocking out P2X4R significantly reduced the proliferation of both PC3 P2X4R KO clones, with a 60% reduction in both KO-1 (*p* = 0.044) and KO-2 (*p* = 0.049) at 96 h, despite only a 12.9% reduction in calcium influx was observed (Fig. 2a). The caspase 3/7 activity apoptosis assay was used to compare the apoptosis between PC3 WT and KO cells. The results demonstrated that knocking out P2X4R significantly enhances the caspase 3/7 activity in both P2X4R KO clones, with a 1.2-fold increase in PC3 KO-1 cells (*p* = 0.1499) and a 3.5-fold increase in PC3 KO-2 cells (*p* = 0.0013) (Fig. 2b). The scratch-wound assay was performed to test the migration ability of PC3 WT/KO cells. The results showed that there were no significant differences in migration among groups, with average areas of migration closure being 37.97% for PC3 WT, 41.31% for PC3 KO-1, and 36.28% for PC3 KO-2, respectively (Fig. 2c and e). Intriguingly, knocking out P2X4R resulted in a significant decrease in invasiveness in both KO-1 and KO-2, with the percentage of invaded area being 76.89% for PC3 WT, 29.84% for PC3 KO-1 (*p* < 0.0001), and 4.57% for PC3 KO-2 (*p* < 0.0001) (Fig. 2d and f).

### P2X4R knockout reduces PCa bone metastasis and bone destruction in vivo

**Fig. 3:**
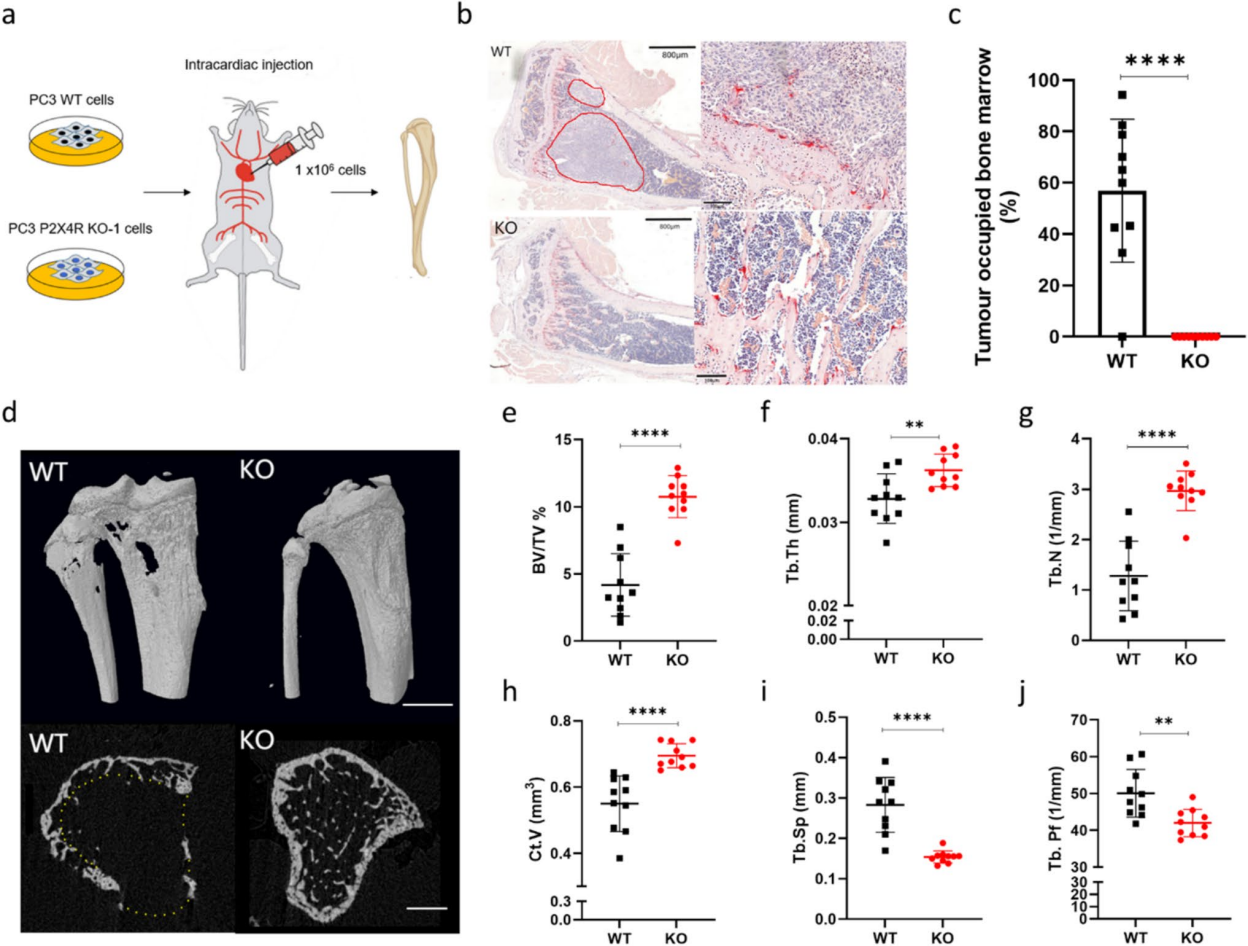
Knocking out P2X4R in PCa cells prevents bone metastasis in vivo. **a** PC3 WT cells or P2X4R KO-1 cells were intracardially injected into 6-week-old BALB/cAnNCrl immunocompromised mice with 1 × 10^5^ cells/injection. Tibias were collected on day 25 for histological and micro-CT analysis ex vivo. **b** Bone marrow area covered by tumour cells in mouse tibial bone were identified on H&E sections. Tumour boundaries were marked in red lines in left-hand panels. Scale bar = 800 μm. Right-hand panels show zoomed in images in affected areas. Scale bar = 100 μm. **c** Percentage area covered by PCa tumour cells were quantified and compared between samples from mice injected with PC3 WT and KO cells. **d** Micro-CT was used to examine bone microarchitecture affected by PC3 tumour in bones. Representative images of 3D models of mouse tibias. Scale bar = 5 cm or 1 cm. **(e-j)** Bone parameters, including trabecular bone volume fraction (BV/TV); trabecular thickness (Tb.Th); trabecular Number (Tb.N); cortical bone volume (Ct.V), trabecular separation (Tb.SP); trabecular pattern factor (Tb. Pf), were quantified and compared between tibias from mice injected with PC3 WT and KO cells. Data are the mean ± SD, *n* = 10, Student’s *t*-test, **p < 0.01, ****p < 0.0001

In the systemic administration intracardiac xenograft mouse model (Fig. 3a), P2X4R-deficient PC3 cells (KO-1) showed a significant reduction in the onset of PCa bone metastasis. Quantifying tumour burden on histology bone sections demonstrated that no overt tumours were present in the mouse tibias injected with KO cells, while on average 53% of the bone marrow area was occupied by WT PC3 tumour (*p* < 0.0001) (Fig. 3b and c).

Micro-CT scanning ex vivo showed that trabecula and cortical bone destruction were observed in tibias of mice injected with PC3 WT cells but not KO cells (Fig. 3d). Further quantitative analysis demonstrated that, compared to tibias from mice injected with KO cells, tibias injected with WT cells had significant lower trabecular bone volume fraction (BV/TV, 61% lower, *p* < 0.0001, Fig. 3e), trabecular thickness (Tb.Th, 9% lower, *p* = 0.0072, Fig. 3f), trabecular number (Tb.N, 57% lower, *p* < 0.0001, Fig. 3g), cortical bone volume (Ct.V, 21% lower, *p* < 0.0001, Fig. 3h), but lower trabecular separation (Tb.Sp, 84% higher, *p* < 0.0001, Fig. 3i) and trabecular pattern factor (Tb.Pf, 19% higher, *p* = 0.0031, Fig. 3j).There were no significant differences in structure model index (SMI) or the degree of anisotropy (DA) (Supplementary Fig. 2a and b).

### Bulk RNA-seq analysis reveals upregulation of anti-invasiveness signalling in KO cells

**Fig. 4:**
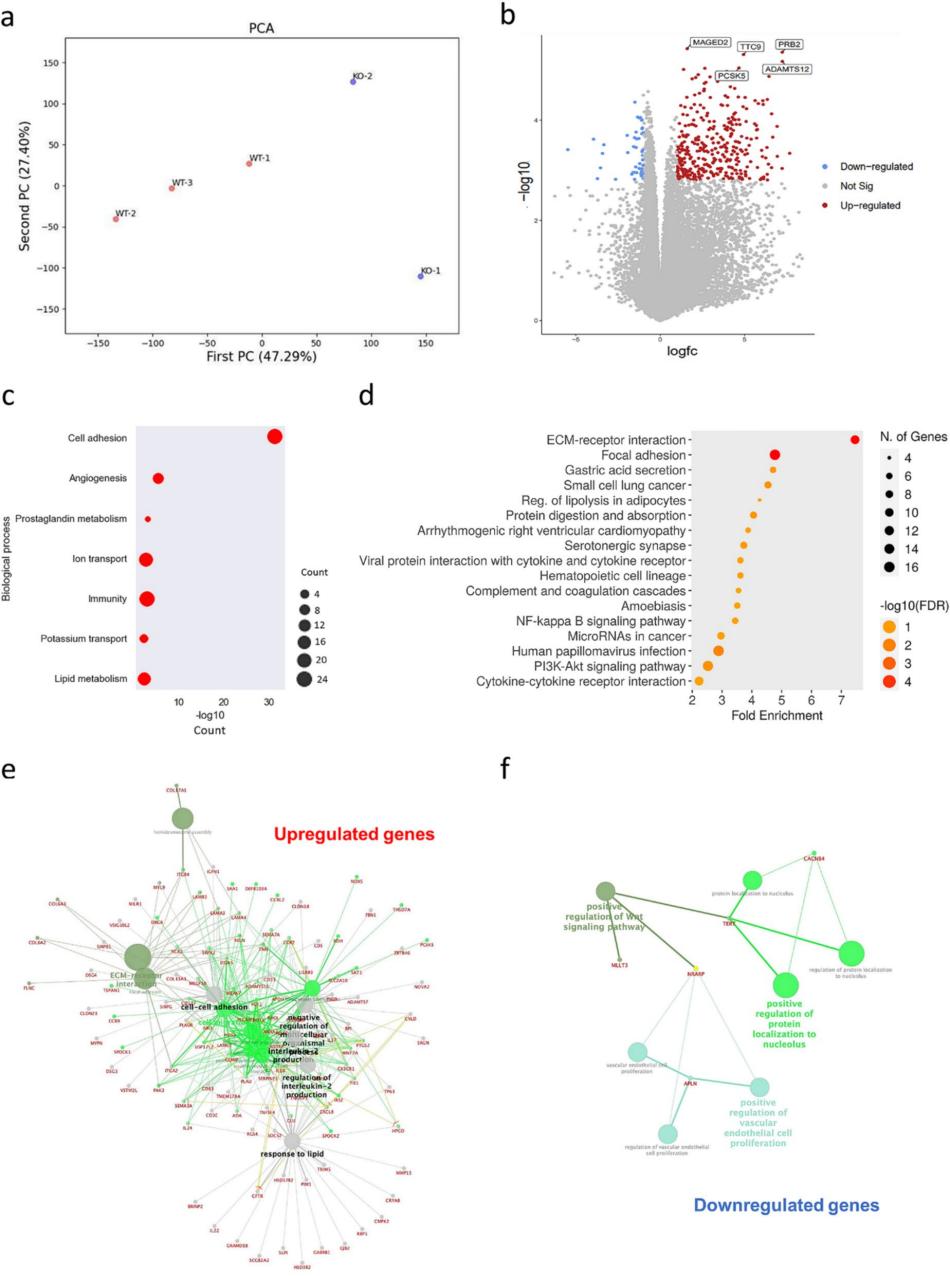
RNA-seq reveals alteration in transcriptome in KO cells. **a** Expression variance between PC3 WT (*n* = 3) and KO cells (KO-1 and KO-2) were conducted on PCA. **b** The volcano plot showed significantly differentially expressed genes in KO cells, with significance defined as logFC ≥ 1 and *p* < 0.05. **c** GO analysis of differentially expressed genes in PCa KO cells showed top altered biological processes. **d** Gene functional annotation shows annotated KEGG pathways. Further gene networking analysis in upregulated genes (**e**) and downregulated genes (**f**) demonstrated enrichments in the regulation of key pathways, including interleukin-2 (IL-2) production, cell–cell adhesion, ECM-receptor interaction, and positive regulation of Wnt signalling pathways

Bulk RNA-seq was performed on both WT and KO cells to reveal P2X4R related genes and pathways in PCa cells. Principal component analysis (PCA) demonstrates considerable variance between PC3 WT replicates and KO clones (Fig. 4a).

The differential gene expression analysis was then performed using the bioinformatics tool Galaxy European to identify 442 significantly upregulated and 45 significantly downregulated genes in KO cells (logFC ≥ 1 and *p* < 0.05) (Fig. 4b). The top ten upregulated and downregulated genes and function are listed in Table 1. Using these 20 genes, further gene correlation analysis via Gene Expression Profiling Interactive Analysis (GEPIA) confirms a negative correlation between *P2RX4* and *IGFN1* (Supplementary Fig. 3a) and a positive correlation between *P2RX4* and *PCDHB7*, *SERPINH1*, and *CACNB4* (Supplementary Fig. 3b-d) in TCGA prostate adenocarcinoma clinical database. GO analysis on differentially expressed genes in PCa KO cells showed cell adhesion (26 genes, 5.3%), angiogenesis (9 genes, 2.0%), and prostaglandin metabolism (3 genes, 0.7%) as the top three enriched biological processes (Table 2, Fig. 4c). Gene functional annotation using David bioinformatics tool suggested that ECM-receptor interactions and focal adhesion are the top two annotated KEGG pathways (Fig. 4e). Further gene networking analysis demonstrates enrichments in regulation of interleukin-2 (IL-2) production, cell–cell adhesion, and ECM-receptor interaction in upregulated genes (Fig. 4e) and positive regulation of Wnt signalling pathways in downregulated genes (Fig. 4f).

**Table 1:** Top 15 upregulated/downregulated genes and functions

	Symbol	Gene name	Log FC	*p*-value
Top 10 upregulated	CLDN18	Claudin 18	7.67	0.04
	ADAMTS12	ADAM metallopeptidase with thrombospondin type 1 motif 12	7.24	0.03
	PRB2	Proline-rich protein BstNI subfamily 2	7.23	0.03
	ST8SIA1	ST8 alpha-N-acetyl-neuraminide alpha-2,8-sialyltransferase 1	7.23	0.04
	BCAS1	Brain-enriched myelin-associated protein 1	6.94	0.04
	SLC16A2	Solute carrier family 16 member 2	6.92	0.03
	CD5	CD5 molecule	6.67	0.03
	PSG9	Pregnancy specific beta-1-glycoprotein 9	6.60	0.03
	ACSM5	Acyl-CoA synthetase medium chain family member 5	6.59	0.04
	IGFN1	Immunoglobulin like and fibronectin type III domain containing 1	6.41	0.04
Top 10 downregulated	COL15A1	Collagen type XV alpha 1 chain	− 5.47	0.03
	PCDHB7	Protocadherin beta 7	− 3.72	0.04
	SERPINH1	Serpin family H member 1	− 3.42	0.03
	MIR611	microRNA 611	− 2.64	0.04
	TEX15	Testis expressed 15, meiosis, and synapsis associated	− 1.95	0.03
	FAM43B	Family with sequence similarity 43 member B	− 1.92	0.03
	KCNK10	Potassium two pore domain channel subfamily K member 10	− 1.68	0.04
	CACNB4	Calcium voltage-gated channel auxiliary subunit beta 4	− 1.61	0.04
	TMEM52	Transmembrane protein 52	− 1.57	0.04
	HIC1	HIC ZBTB transcriptional repressor 1	− 1.54	0.04

**Table 2:** Gene ontology enrichment of biological process

Biological process	Related genes	Count	%	*p*-value
Cell adhesion	CLDN18,ADAMTS12,CD177,CD33,NUAK1,ADA,APLP1,COL6A2,COL6A3,COL13A1,COL15A1,DSG3,DSG4,ITGA2,ITGA5,ITGB4,LAMA3,LAMA4,LAMB3,LAMC2,MEGF10,PECAM1,PCDHB7,RELN,SIRPG,SRPX2	26	5.3	0.00001
Angiogenesis	NOX5,APLN,COL15A1,FGF1,PDE3B,RHOJ,SRPX2,THSD7A,TIE1	9	2.0	0.00287
Prostaglandin metabolism	HPGD,PTGS1,PTGS2	3	0.7	0.02083
Ion transport	ATP1B4,CFTR,NOX5,CACHD1,CACNB4,CACNA1A,GABRB1,GRIK2,HEPHL1,KCTD12,KCNJ15,KCNJ9,KCNK10,KCNG1,KCND1,RYR2,SLC24A5,SLC4A8,SLC5A7,SLC9A4	20	4.4	0.02180
Immunity	CX3CR1,CD177,CD3E,CYLD,FCGR1BP,FCMR,TRAV13-2,TRAV30,TRAV39,TRAV8-4,TRBJ2-3,TRBJ2-7,TXK,ADGRE1,BPI,CLU,C1S,GBP1,IDO2,LILRB3,MARCO,OPTN,SLPI,SAMD9,TRIM5	25	5.5	0.02690

## Discussion

The involvement of P2X4R in tumorigenesis has been demonstrated in breast cancer, gastric cancer, and PCa [8–10, 12]. In this study, we show that P2X4R deficiency impairs PCa cell proliferation and invasiveness in vitro*,* and leads to the inhibition of overt bone metastases in vivo. Our results are consistent with previous research and, more importantly, provide insights for understanding mechanisms of action by which P2X4R affects PCa biology, especially PCa bone metastasis.

One of the possible mechanisms is that silencing P2X4 might affect the expression of other P2X receptors subtypes. To test this hypothesis, we have performed quantitative real-time PCR and examined the transcriptional expression of all P2X receptors in WT and both PC3 P2X4R KO clones. The results suggest that silencing P2X4R does not lead to significant changes in the expression of other P2X receptors (Supplementary Fig. 4a–f), indicating that the biological consequence of P2X4R KO is not due to redundancy or over-compensation by other P2X receptor subtypes.

The RNA-seq analysis revealed that the deficiency of P2X4R may negatively impact PCa metastasis through upregulating cell–cell adhesion and modulating cancer cell invasion. Previous evidence demonstrated that cell–cell adhesion, and extracellular matrix (ECM)-related adhesion proteins play a vital role in regulating various stages of metastasis and defining the aggressiveness of cancer cells [15, 16]. During cancer invasion and metastasis, the loss of cell–cell adhesion is a key step as adherens junctions are crucial for maintaining epithelial homeostasis and regulating cancer cell migration and tumour progression [17, 18]. The loss of cell–cell adhesion allows changes in cell–matrix interaction, enabling cells to invade the surrounding stroma during the invasion process [19]. Additionally, gene functional annotation on our data set suggested enriched ECM-receptor interactions and focal adhesion which all play crucial roles in cell adhesion activity and reduce cancer cell invasiveness [20]. The enhanced cell–cell adhesion, increased ECM-receptor interactions and focal adhesion in P2X4R KO cells can largely explain our observations of reduced invasiveness but no changes in migration in vitro, and significant reductions in PCa bone metastasis in vivo. This is consistent with the findings from our previous study and Maynard et al. that P2X4R antagonism e.g. by selective P2X4R antagonist 5-BDBD, resulted in reduced invasion of various PCa cell lines [11, 12]. At the molecular level, this enhanced cell adhesion could be attributed to the deregulated WNT-β-catenin signalling and other signals/genes e.g. the top 2 upregulated genes CLDN18 and ADAMTS12, which have all been implicated in modulating cell adhesion activities [21–23]. However, how P2X4R interacts or regulates these signalling pathways and genes needs further investigation.

Intriguingly, our RNA-seq analysis on P2X4R KO cells also suggested an upregulation of IL-2 production. However, it is well documented that PC3 cells do not secret IL-2 [24] and our gene correlation analysis using GEPIA also confirms that there is no gene expression correlation between *P2RX4* and *IL-2* in prostate adenocarcinoma (Supplementary Fig. 3e) but a positive correlation in whole blood (Supplementary Fig. 3f). Although our RNA-seq data appears contradictory to this evidence, it still provides insights into the potential link between P2X4R and IL-2. Currently, direct evidence linking P2X4R specifically to IL-2 regulation is limited and remains T-cell focused. For example, P2X4R was shown to positively contribute to NFAT activation and IL-2 expression in T-cells at the immune synapse [25]. Our data demonstrated that P2X4R may play an indirect regulatory role in modulating IL-2 production at cellular level. This highlights the need for a comprehensive investigation into the systemic effects of P2X4R-targeting therapies in PCa treatment, particularly regarding immune response. Furthermore, the applications of P2X4R targeting therapies have been primarily investigated for their involvement in neuropathic and inflammatory pain [26]. Pharmacological studies have identified P2X4R antagonists as potential therapeutic agents. For instance, the P2X4R-selective antagonist NP-1815-PX has demonstrated anti-allodynic effects in chronic pain mouse models [27]. No side effects have been identified for pharmacologically targeting P2X4R in breast cancer [9] or PCa [11, 12] in vivo. This provides confidence in the future clinical application of P2X4R targeting therapies.

While this study employed both in vitro and in vivo models, it has several limitations, including exclusive use of a single prostate cancer cell line (PC3) and a single knockout (KO) clone (KO-1) in the in vivo experiments. Verification with additional cell lines and KO clones is necessary to account for cell line–specific characteristics and clonal variability, and to avoid potential over- or underestimation of the effects of P2X4R deletion. Another limitation is the reliance on a xenograft model, which lacks an intact immune system and therefore limits our ability to fully assess the role of P2X4R silencing in prostate cancer cells in vivo. Further in vivo studies using selective antagonists to pharmacologically inhibit P2X4R would strengthen the evidence for its therapeutic potential in reducing prostate cancer bone metastasis. In particular, combining these approaches with syngeneic models could provide critical insights into the effects of P2X4R-targeting therapies on the tumour microenvironment and immune responses, such as IL-2 production. Finally, validation of the targets identified in P2X4R KO cells at the protein levels will further elaborate mechanisms involved in P2X4R regulated prostate cancer biology.

In conclusion, our study provides further exciting evidence for the involvement of P2X4R in the aggressiveness of PCa, particularly in the context of bone metastasis. Knocking out P2X4R in PC3 cells impairs tumour cell invasiveness and leads to the inhibition of PCa bone metastasis in vivo. These findings suggest that P2X4R could serve as a potential therapeutic target in treating metastatic PCa.

## Supplementary Information

Below is the link to the electronic supplementary material.

ESM 1 (PNG 1.90 MB)

High Resolution Image (TIF 16.1 MB)

ESM 2 (PNG 239 KB)

High Resolution Image (TIF 7.19 MB)

ESM 3 (PNG 1.35 MB)

High Resolution Image (TIF 1.87 MB)

ESM 4 (PNG 427 KB)

High Resolution Image (TIF 502 KB)

## Data Availability

RNA-seq data can be accessed on the GEO repository (submission GSE245125). Other datasets generated during the current study are available from the corresponding author upon reasonable request.
