# Supplementary figures and images for: Depletion of P2X4 receptor alleviates prostate cancer bone metastasis through reduced cancer cell invasiveness and enhanced cell adhesion activities
Source: Purinergic Signal. 2025 Jun 14;21(5):1065–75. doi: 10.1007/s11302-025-10096-5 (PMC12595180; doi:10.1007/s11302-025-10096-5)

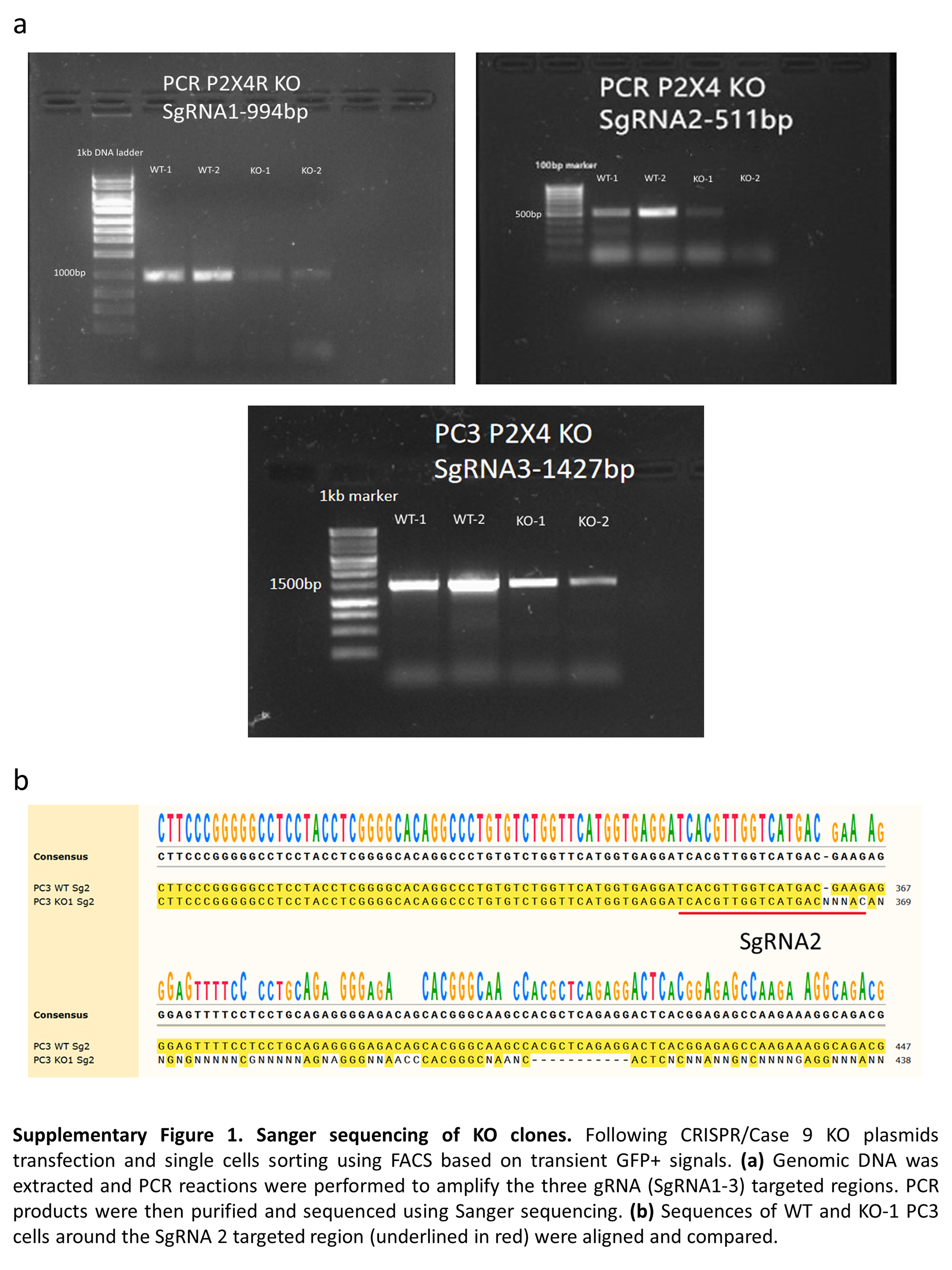

Supplement: Supplementary file 1 — (PNG 1.90 MB) [file 11302_2025_10096_Fig5_ESM.png]

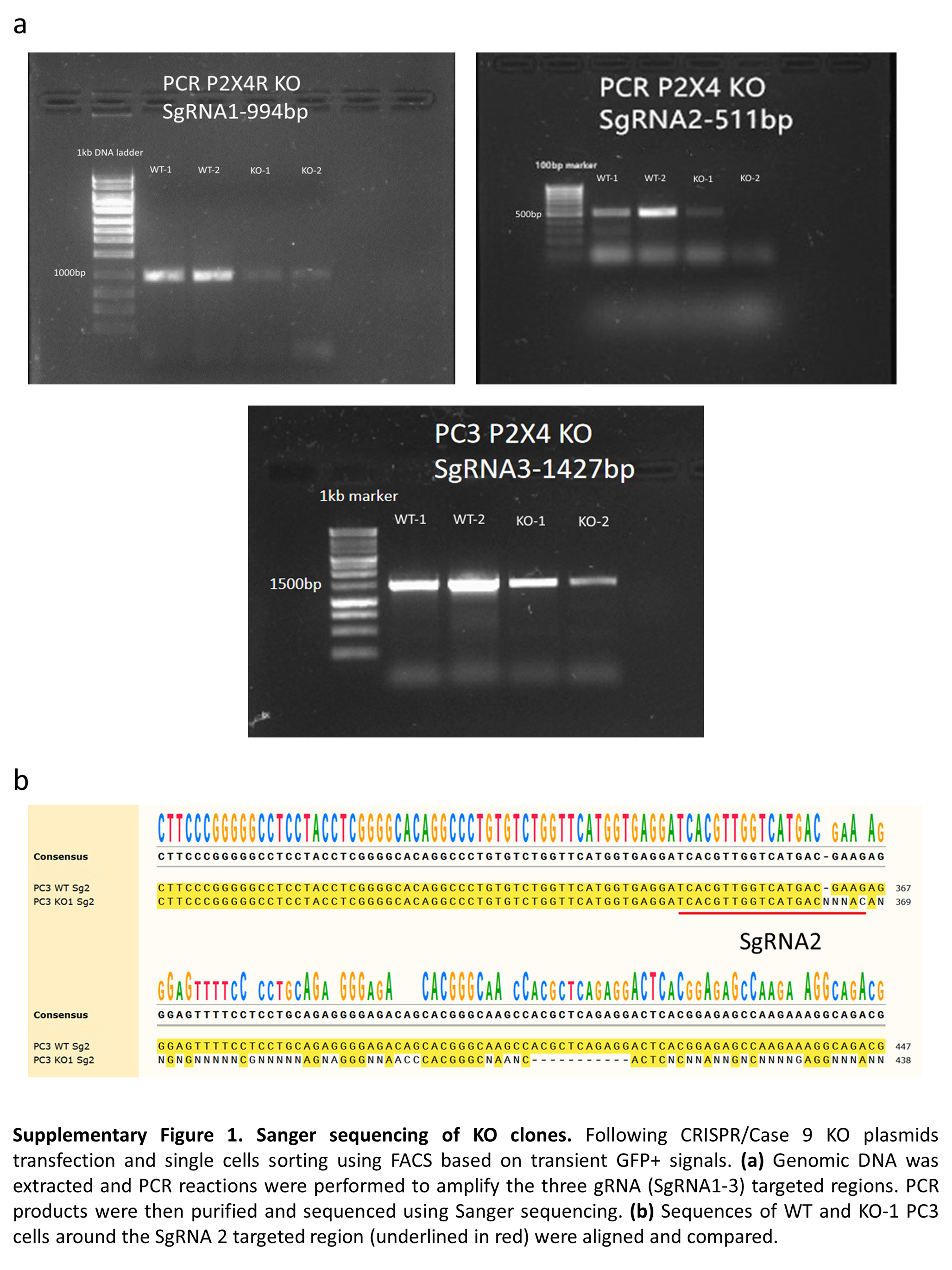

Supplement: Supplementary file 2 — High Resolution Image (TIF 16.1 MB) [file 11302_2025_10096_MOESM1_ESM.tif]

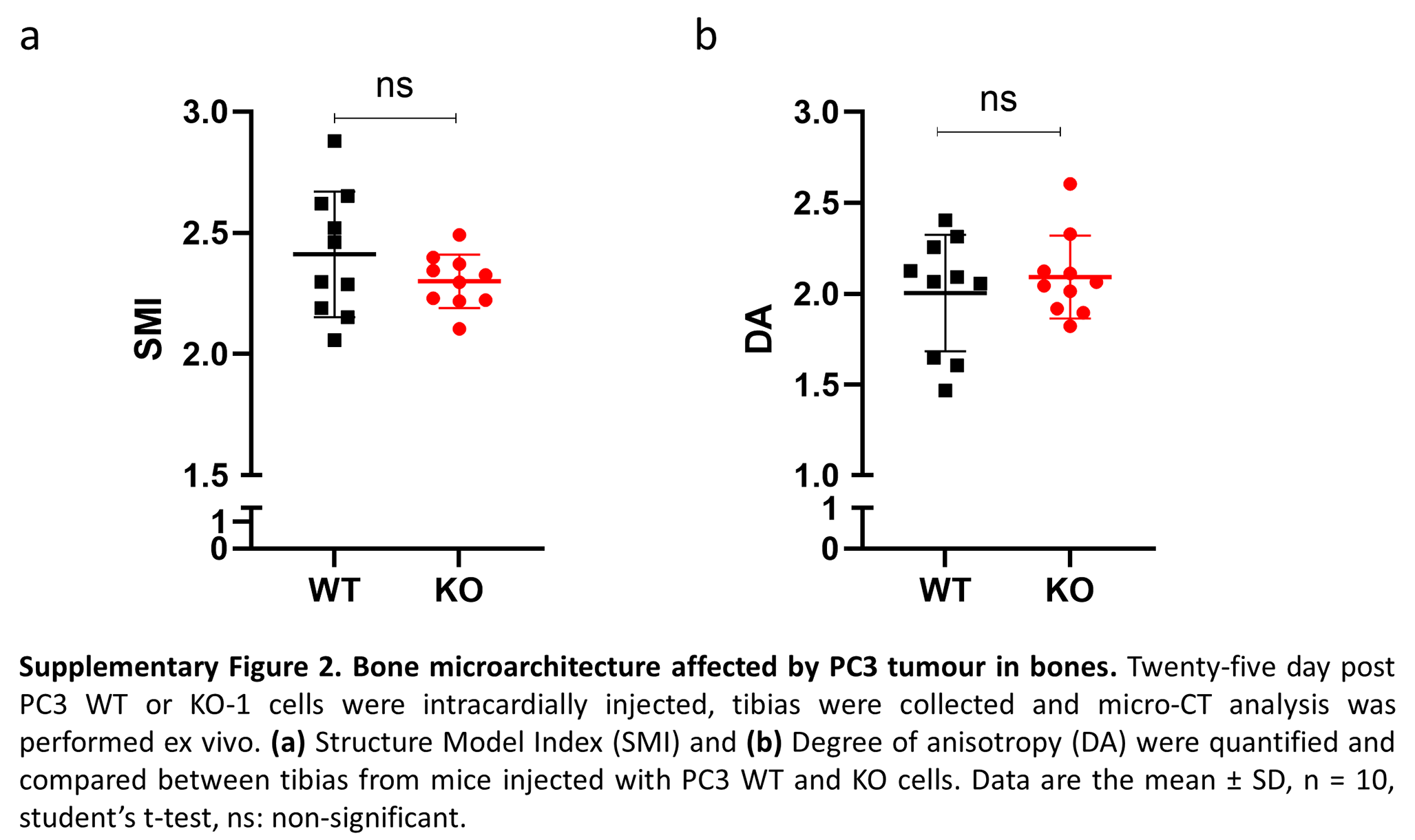

Supplement: Supplementary file 3 — (PNG 239 KB) [file 11302_2025_10096_Fig6_ESM.png]

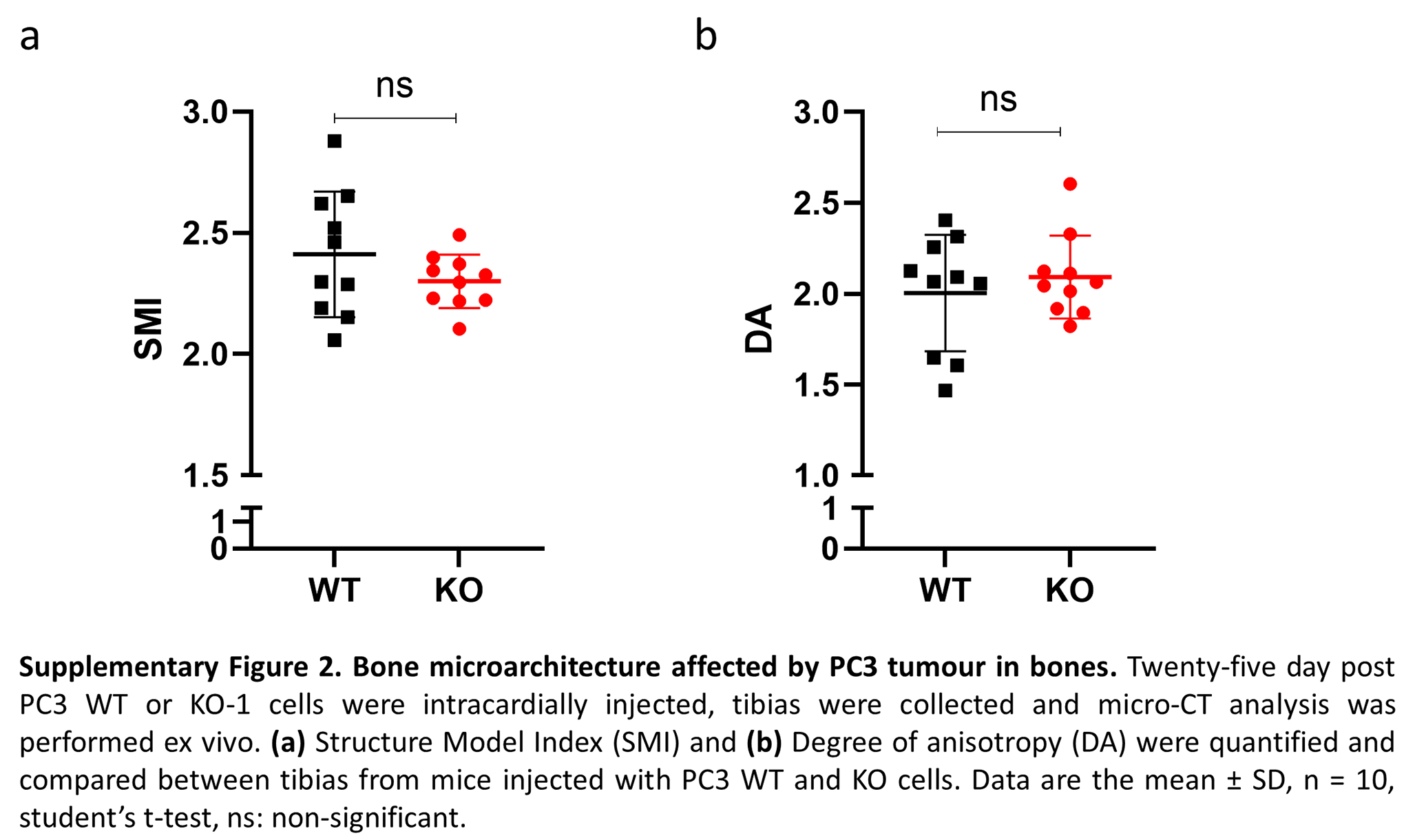

Supplement: Supplementary file 4 — High Resolution Image (TIF 7.19 MB) [file 11302_2025_10096_MOESM2_ESM.tif]

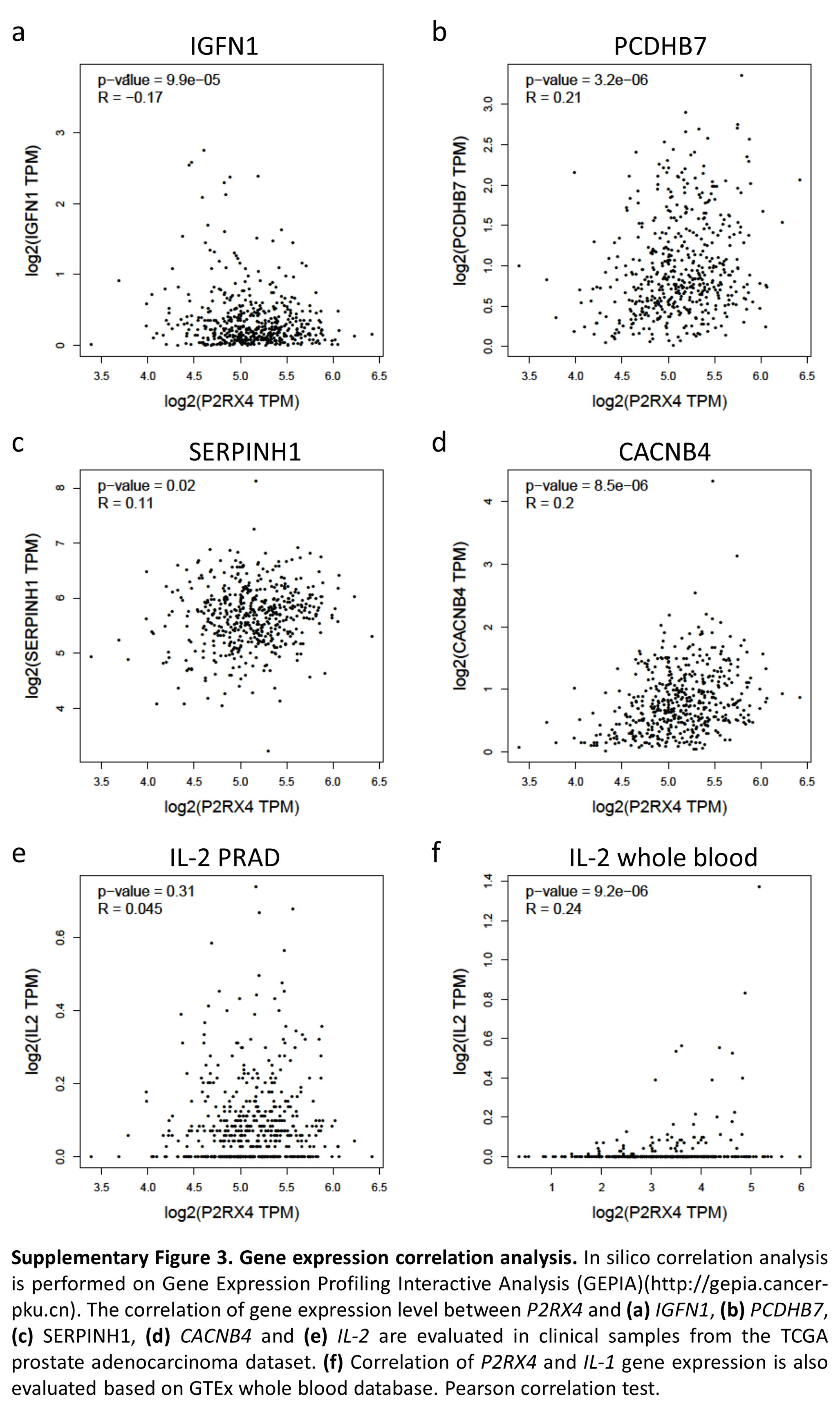

Supplement: Supplementary file 5 — (PNG 1.35 MB) [file 11302_2025_10096_Fig7_ESM.png]

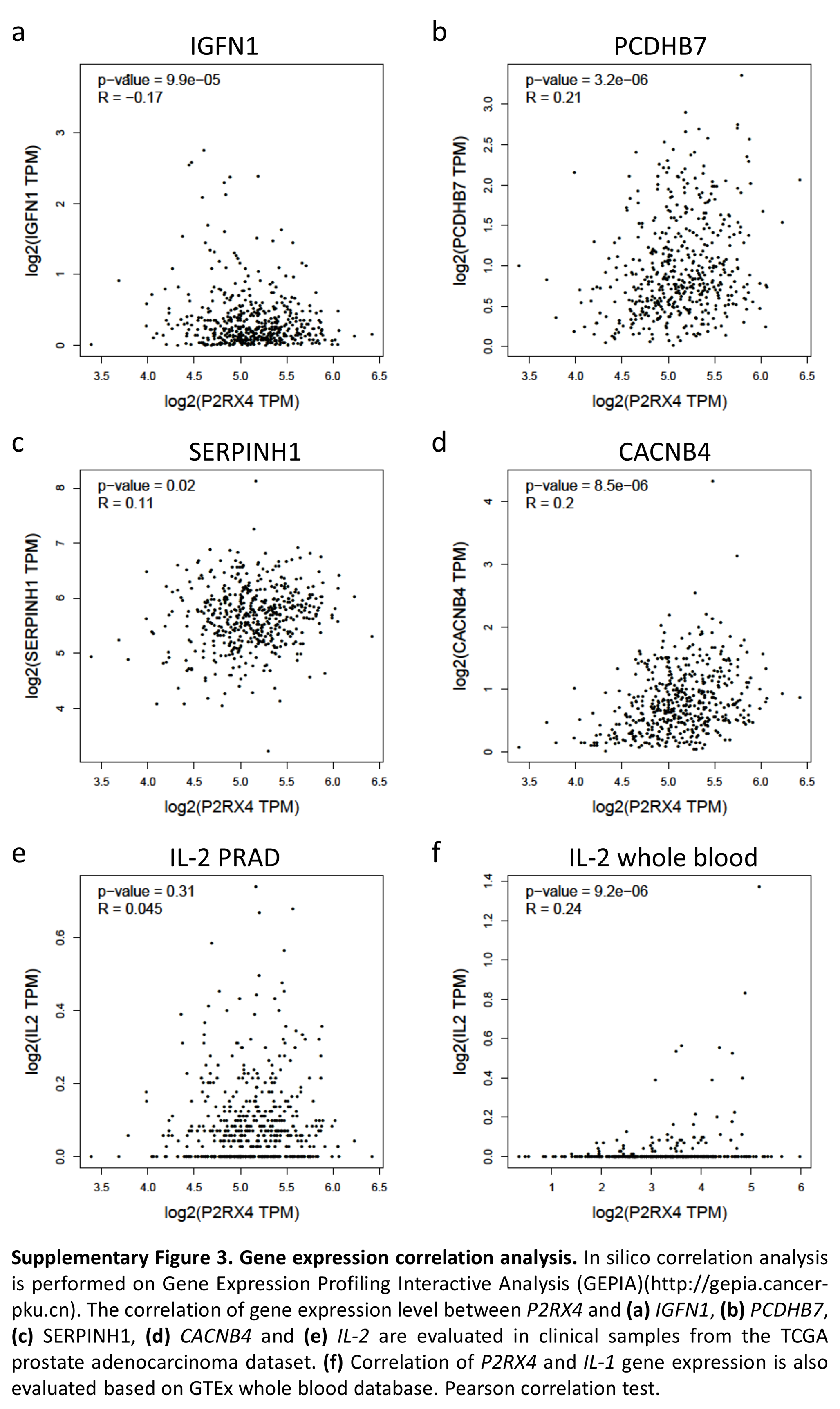

Supplement: Supplementary file 6 — High Resolution Image (TIF 1.87 MB) [file 11302_2025_10096_MOESM3_ESM.tif]

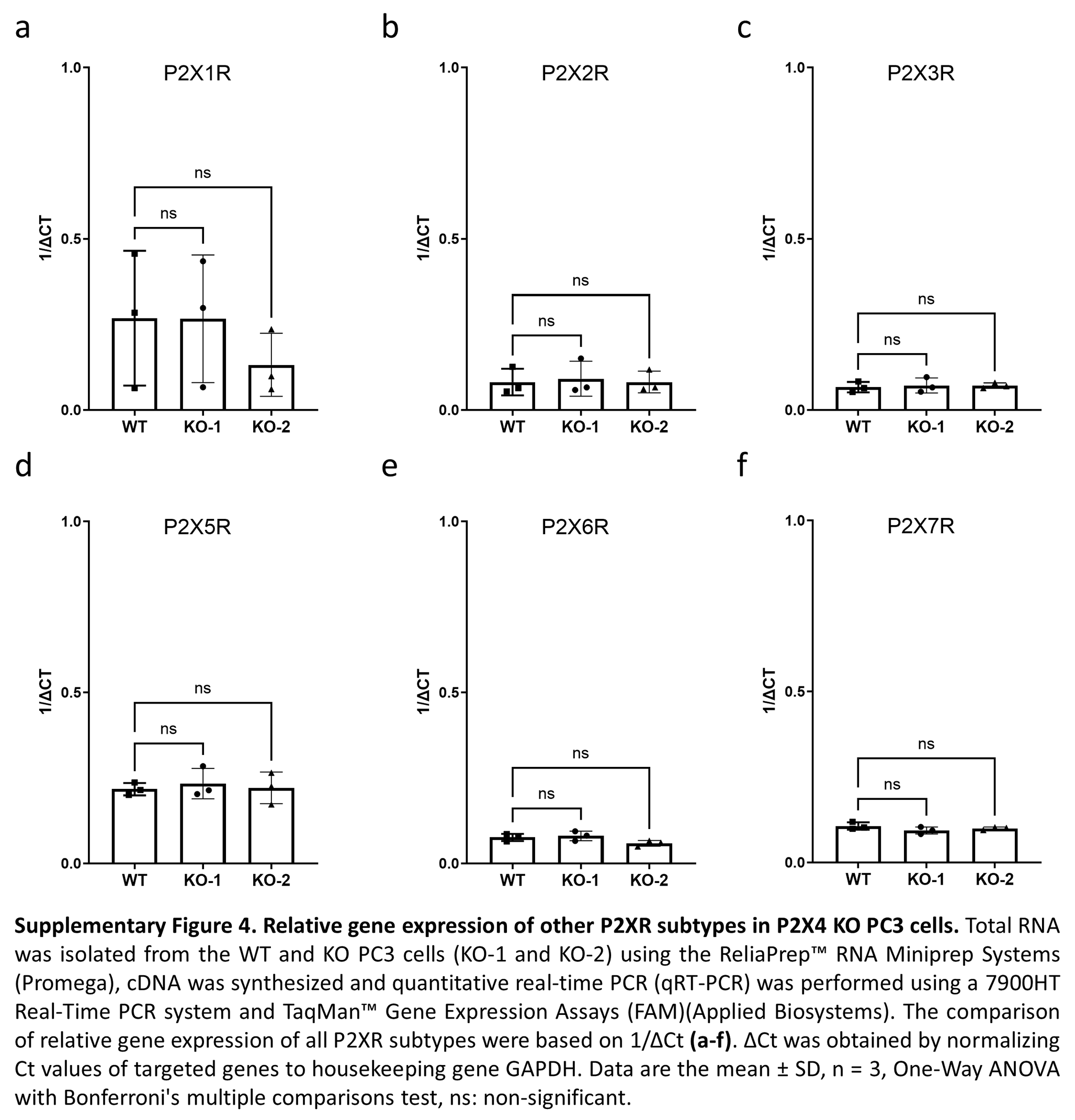

Supplement: Supplementary file 7 — (PNG 427 KB) [file 11302_2025_10096_Fig8_ESM.png]

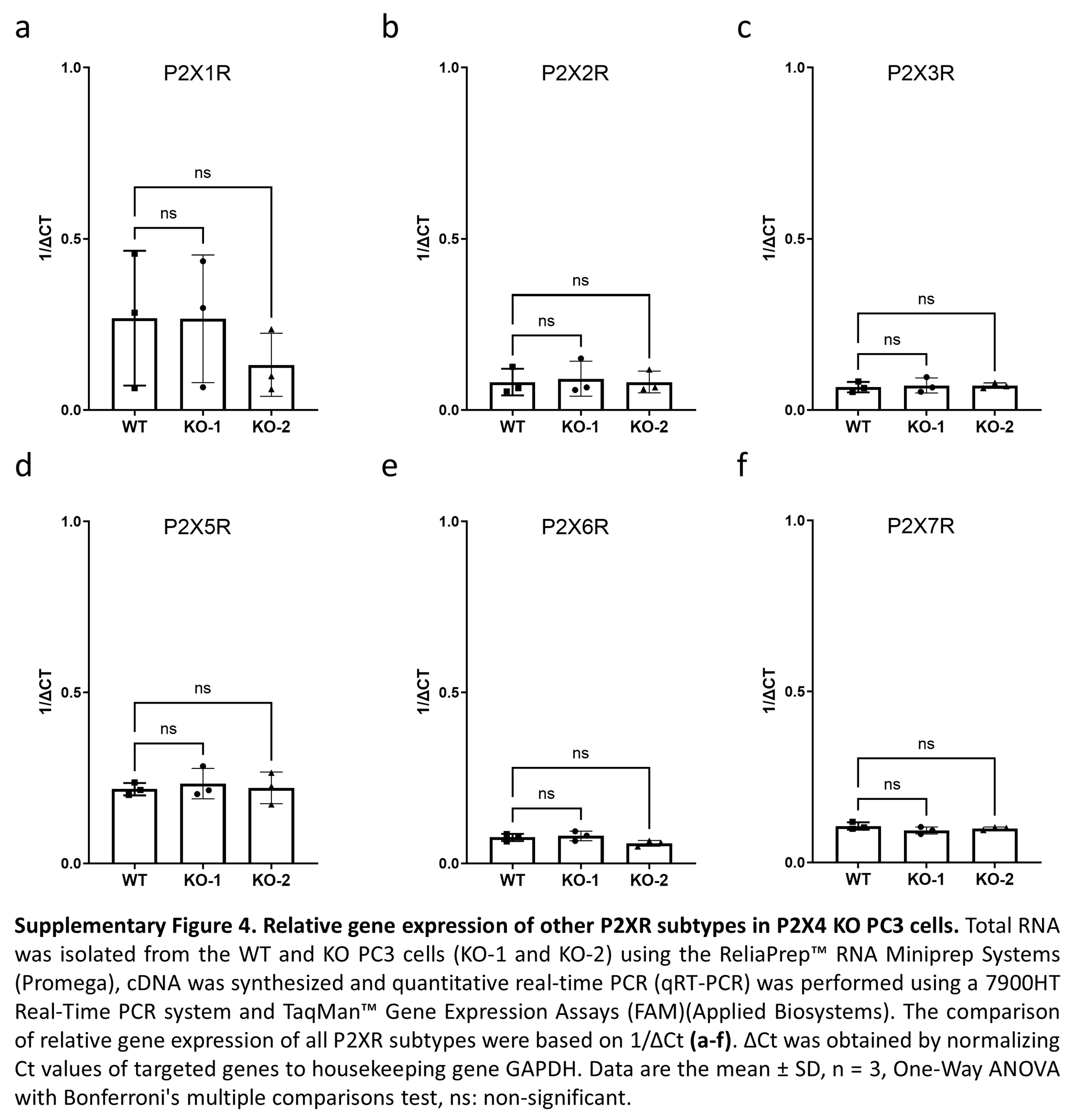

Supplement: Supplementary file 8 — High Resolution Image (TIF 502 KB) [file 11302_2025_10096_MOESM4_ESM.tif]
